# 7 Day Follow-Up Arrangements Following Discharge From Psychiatric Hospital; How Do We Perform?

**DOI:** 10.1192/bjo.2022.407

**Published:** 2022-06-20

**Authors:** Jemima Alston, Rozet Balliou, Naomi Erlebach, Zach Evans, Anna Grieve, Silas Hand, Shreya Jindal, Phei Yi Lim, Matt Shaw, Chukyi Wama, Ben Meadowcroft, Douglas Murdie

**Affiliations:** 1 University of Edinburgh, Edinburgh, United Kingdom; 2 NHS Lothian, Edinburgh, United Kingdom

## Abstract

**Aims:**

The first 7 days following discharge from inpatient to community psychiatric services is a period that is associated with an increased risk of suicide. NICE Guideline 53 recommends that patients discharged from inpatient psychiatric services should be reviewed by relevant community services within 7 days. We aim to determine how different teams in NHS Lothian performed in meeting this recommendation, and to ascertain the outcome of a specific intervention in North-West Edinburgh (NW).

**Methods:**

We collected data of NW, North-East (NE), South-West (SW), South-East (SE) Edinburgh, East Lothian and Midlothian patients discharged from General Adult Psychiatry wards in the Royal Edinburgh Hospital for the calendar year of 2021. East and Midlothian were used as a comparison to Edinburgh services as the former have an integrated inpatient and community team.

The data focused on the percentage of patients followed-up within 7 days of discharge. We also collected data for all NW CMHT patients discharged between January 2018 and November 2021 to analyse the intervention of using ‘Estimated Discharge Dates’ in ward rounds implemented in June 2020. Data were collected from NHS Lothian Analytical Services and anonymised in line with NHS Information Governance Policy.

Furthermore, qualitative data were collected anonymously from staff within NHS Lothian in the form of an online questionnaire to ascertain strengths and weaknesses of the current systems.

**Results:**

Over the calendar year of 2021, 1,398 patients were discharged. The average age was 41 years old.

Regarding percentage of patients receiving 7 day follow-up, East Lothian (n = 191/249; 76.7%) and Midlothian (n = 95/122; 77.9%) performed better than Edinburgh services; NW (n = 173/268; 64.6%), NE (n = 172/301, 57.1%), SW (n = 155/247, 62.8%), SE (n = 123/211; 58.3%).

The intervention in NW in June 2020 did not have a significant impact on 7 day follow-up.

The questionnaire identified difficulties in transitions from inpatient to community care, particularly communication between teams.

**Conclusion:**

The performance of East and Midlothian versus Edinburgh services is interesting given their integrated model. This appears to support the findings of the questionnaire.

The lack of impact of the intervention in NW will need explored further with the team to identify difficulties.

Rather than complete service remodelling, perhaps moving towards a more integrated approach such as allocated discharge-coordinating community and inpatient nurses would be worthwhile. We will involve the NHS Lothian Quality Improvement team in exploring this to improve patient outcomes.

